# Reward Boosts Neural Coding of Task Rules to Optimize Cognitive Flexibility

**DOI:** 10.1523/JNEUROSCI.0631-19.2019

**Published:** 2019-10-23

**Authors:** Sam Hall-McMaster, Paul S. Muhle-Karbe, Nicholas E. Myers, Mark G. Stokes

**Affiliations:** ^1^Department of Experimental Psychology, University of Oxford, Oxford OX2 6AE, United Kingdom, and; ^2^Wellcome Centre for Integrative Neuroimaging, University of Oxford, Oxford OX3 9DU, United Kingdom

**Keywords:** cognitive control, flexibility, motivation, representational similarity analysis, reward prospect

## Abstract

Cognitive flexibility is critical for intelligent behavior. However, its execution is effortful and often suboptimal. Recent work indicates that flexible behavior can be improved by the prospect of reward, which suggests that rewards optimize flexible control processes. Here we investigated how different reward prospects influence neural encoding of task rule information to optimize cognitive flexibility. We applied representational similarity analysis to human electroencephalograms, recorded while female and male participants performed a rule-guided decision-making task. During the task, the prospect of reward varied from trial to trial. Participants made faster, more accurate judgements on high-reward trials. Critically, high reward boosted neural coding of the active task rule, and the extent of this increase was associated with improvements in task performance. Additionally, the effect of high reward on task rule coding was most pronounced on switch trials, where rules were updated relative to the previous trial. These results suggest that reward prospect can promote cognitive performance by strengthening neural coding of task rule information, helping to improve cognitive flexibility during complex behavior.

**SIGNIFICANCE STATEMENT** The importance of motivation is evident in the ubiquity with which reward prospect guides adaptive behavior and the striking number of neurological conditions associated with motivational impairments. In this study, we investigated how dynamic changes in motivation, as manipulated through reward, shape neural coding for task rules during a flexible decision-making task. The results of this work suggest that motivation to obtain reward modulates the encoding of task rules needed for flexible behavior. The extent to which reward increased task rule coding also tracked improvements in behavioral performance under high-reward conditions. These findings help to inform how motivation shapes neural processing in the healthy human brain.

## Introduction

Flexible cognitive control is critical to human intelligence. When vying to win a card game, we can use arbitrary rules to play the best hand. When navigating a new city, we can apply navigation rules to sensory input from the world around us to arrive at the next tourist attraction. Controlled processes requiring flexibility result in slower performance, compared with behaviors that require less flexible processing (Shen and Chun, 2011; Kleinsorge and Rinkenauer, 2012). However, this can be improved by motivational factors, such as the prospect of reward for speed and accuracy (Braem and Egner, 2018). When the prospect of reward is high, performance improves on flexible rule-based tasks (Kleinsorge and Rinkenauer, 2012; Etzel et al., 2016), suggesting that reward might optimize cognitive flexibility by fine-tuning rule-based neural coding patterns.

The existing neuroimaging literature provides support for this perspective, indicating that reward prospect leads to stronger recruitment of frontoparietal brain regions implicated in cognitive control (Parro et al., 2018). When reward cues are presented at the beginning of each trial, activity in these regions is typically enhanced before the onset of a target stimulus, suggesting that proactive control mechanisms contribute to reward-induced performance benefits (Engelmann et al., 2009; Padmala and Pessoa, 2011; Krebs et al., 2012). Critically, a recent study combining fMRI with pattern classification methods found that frontoparietal activity before target onset encoded abstract task rules with greater fidelity on reward trials than on no-reward trials (Etzel et al., 2016). The extent to which reward prospect enhanced the decodability of task rules also mediated behavioral benefits, suggesting that performance improvements may result from reward-driven tuning in cognitive control processes that prioritize task-relevant processing.

This proposal has considerable theoretical appeal. Yet, it is unknown whether reward prospect differentially influences rule coding in situations that require more or less flexible processing. The present study therefore aimed to characterize how changes in reward influence neural coding of task rule information when rules must be updated. To do so, we developed a behavioral task that involved switching between task rules, as reward prospect was manipulated. We then applied representational similarity analysis (RSA; Kriegeskorte et al., 2008) to human electroencephalograms (EEGs) to examine changes in neural coding as a function of reward prospect. Based on the findings of Etzel et al. (2016), we predicted that task rule coding would be greater with high reward prospect and that the extent of this increase would track improvements in behavioral performance. However, the critical contribution of this work was to consider how reward prospect would impact rule coding when flexible processing was needed. Based on prior work showing selective reward enhancements on rule switching (Shen and Chun, 2011; Kleinsorge and Rinkenauer, 2012), we reasoned that task rule coding should be greatest on high-reward switch trials, where task rules must be updated. Switch trials involve the most interference between rule codes, and thus increased neural separation between them should support flexible rule updating (Waskom et al., 2014). Reward prospect could therefore optimize flexible processing by helping to increase neural dissimilarity between rules, when they come into conflict. RSA is well suited to test this prediction because it provides a sensitive index of reward-driven changes in neural dissimilarity, while allowing clean separation of overlapping task variables in the lead up to adaptive behavioral responses.

To summarize the main results, we found that high reward prospect produced significant performance improvements in accuracy and reaction time (RT). Consistent with the view that reward prospect increases proactive cognitive control, we found a significant increase in neural coding for task rules under high-reward conditions before the onset of a target stimulus. The average difference in rule coding between reward conditions during this period also correlated with RT improvements. Especially striking, the effect of reward on rule coding was most pronounced in situations requiring the most flexible processing, when task rules were updated relative to the previous trial.

## Materials and Methods

### 

#### Participants

We set a target sample size of 30 participants. During recruitment, three participants were excluded, one due to a corrupt EEG recording and two due to excessive artifacts that led to the rejection of >120 of 650 trials. We therefore collected three more participants to reach the 30 participant target. The final sample size was composed of participants between 18 and 35 years of age (mean age, 23 years; 19 females), with normal or corrected-to-normal vision, who reported no history of neurological or psychiatric illness. Participants received £8/h or course credit for taking part and could earn up to £10 extra for their performance. This study was approved by the Central University Research Ethics Committee at the University of Oxford, and all participants signed an informed consent form before taking part.

#### Materials

Stimuli were presented on a 22 inch screen with a spatial resolution of 1280 × 1024 and a refresh rate of 60 Hz. Stimulus presentation was controlled using Psychophysics Toolbox-3 (RRID:SCR_002881; Kleiner et al., 2007) in MATLAB (version R2015b; RRID:SCR_001622). Reward cues and feedback shown during the task were presented in size 30 Arial font. Task cues and target stimuli had approximate visual angles of 2.52° (100 × 100 pixels) and 1.26° (50 × 50 pixels), respectively, with visual angles calculated based on an approximate viewing distance of 60 cm. F and J keys on a standard QWERTY keyboard were used to record left and right hand responses. EEG data were recorded with 61 Ag/AgCl sintered electrodes (EasyCap), a NeuroScan SynAmps RT amplifier, and Curry 7 acquisition software (RRID:SCR_009546). EEG data were preprocessed in EEGLAB (version 14.1.1b; RRID:SCR_007292; Delorme and Makeig, 2004), behavioral analyses were performed using JASP (version 0.8.1.3; RRID:SCR_015823), and EEG analyses were performed in MATLAB (version 2018b), using the FieldTrip toolbox (RRID:SCR_004849) as well as custom scripts.

#### Code accessibility

Task and analysis code, as well as raw and preprocessed data can be accessed at: https://osf.io/kuzye/.

#### Experimental design and statistical analysis

**Figure 1.**
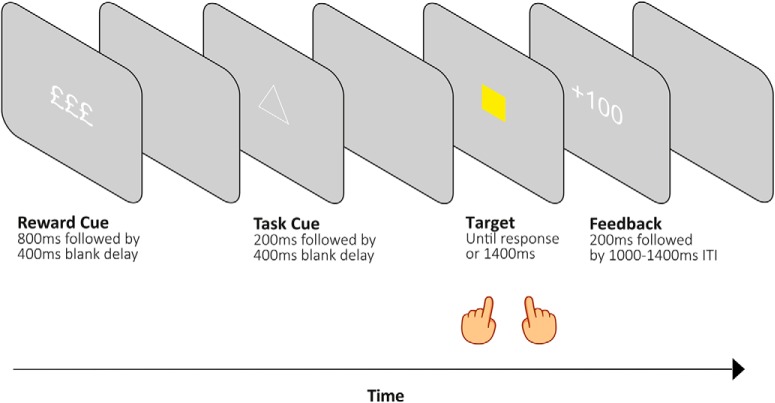
Task design. On each trial, a high-reward or low-reward cue was presented followed by a blank delay. A task rule cue was then presented, indicating whether participants should respond to the upcoming target based on its color or shape. Following a second blank delay, a bidimensional target (a colored shape) appeared until a response was given or for a maximum duration of 1400 ms. This was followed by feedback (based on accuracy and reaction time) and a variable intertrial interval.

In this task (Fig. 1), participants' overarching goal was to gain as many points as possible. To do so, participants categorized bidimensional target stimuli based on their color (yellow vs blue) or shape (square vs circle). On each trial, only one feature dimension of the target (color or shape) was relevant to gaining points, while the other feature served as an irrelevant distractor. The relevant feature dimension was signaled through a visual task cue before target onset. In addition to a single relevant feature dimension, each trial offered a high or low reward magnitude for making a correct response. This was signaled to participants at the beginning of each trial by a single pound sign (£; low reward, 5–10 points) or three pound signs (£££; high reward, 50–100 points).

The experimental sequence consisted of a reward cue, task cue, target, feedback screen, and an intertrial interval (ITI). The reward cue (£ or £££) was first presented for 800 ms, followed by a 400 ms delay. The task cue (one of four possible abstract shapes) was then presented for 200 ms. The mapping of cues to tasks was counterbalanced between participants. Task cue offset was followed by a 400 ms delay. The target (a yellow square, blue square, yellow circle, or blue circle) was then presented and remained on screen until a response was given or for a maximum of 1400 ms. If the active task rule was color, the correct response mapping was “f” or “j” for yellow or blue targets, respectively. If the active rule was shape, the correct response mapping was “f” or “j” for square or circle targets, respectively. The response phase was followed by feedback lasting 200 ms. An incorrect response or omission resulted in feedback showing “+0” points. A correct response resulted in feedback showing “+X,” where X was a value within the high or low reward point ranges, the precise value of which was determined by RT. More specifically, RT criteria for different points were initialized so that responses faster than 400, 600, 800, 1000, 1200, and 1400 ms earned 100/10, 90/9, 80/8, 70/7, 60/6, and 50/5 points on high and low reward trials, respectively. For correct trials, the current trial RT was added to an array for its reward condition. When each array contained more than six values, individualized points criteria were calculated for that condition and were calculated again every time a new value entered the array. The individualized points criteria followed criteria outlined by Shen and Chun (2011), in which the most (to least) points are rewarded for correct responses faster than 95%, 80%, 65%, 50%, and 35% of previous condition RTs. The trial concluded with a randomly selected ITI duration, drawn from a uniform distribution with values of 1000, 1100, 1200, 1300, or 1400 ms. Participants were trained to reach a criterion of 70% accuracy before completing 10 experimental blocks of 65 trials. Excluding the first trial in each block, equal numbers of reward cues, task cues, stimuli, and ITI durations were presented. Presentation was pseudorandomized to ensure that trials were balanced based on task, target congruency, and task sequence for each reward condition. Target congruency refers to whether task-relevant and task-irrelevant target features are mapped to the same (congruent) or different (incongruent) response hands. Task sequence refers to whether the task rule was different from the previous trial (switch trial) or the same as the previous trial (repeat trial).

##### Behavioral measures.

Our main dependent measures were RT and the proportion of correct responses (accuracy). We calculated median RTs for the respective design cells to account for the skewness of RT distributions (Ratcliff, 1979). For all RT analyses, we only included trials in which the current and previous trials were correct to mitigate potential effects of posterror slowing, in which participants tend to respond more slowly after making an error (Notebaert et al., 2009; Dutilh et al., 2012). For accuracy analyses, we included all trials in which a response was made within the 1.4 s response window. Behavioral data were analyzed using 2 × 2 repeated-measures ANOVAs with factors of reward and task sequence. The task sequence factor consisted of switch trials, where the task rule was different from the previous trial, and repeat trials, where the task rule was the same as the previous trial. In addition, we used a paired-sample *t* test for normally distributed data and a Wilcoxon signed-rank test for data showing significant deviation from normality to compare performance on task-switch trials and cue-switch trials. This analysis served as a behavioral control. It has been argued that switch costs could arise simply from processes related to processing the cue stimulus, rather than changes in the task rule per se (Logan and Bundesen, 2003; Logan and Schneider, 2006). Comparing trials where both task cue and task rule changed, with trials where the cue changed but the rule stayed the same allowed us to delineate the extent to which switch costs were driven by changes in task rules and task cues. More specifically, if the effect of rule switching on behavioral performance was primarily driven by rule changes, we should see greater performance reductions on trials where the rule is changed relative to trials where the rule is repeated but the cue is changed.

##### EEG preprocessing.

EEG data were down-sampled from 1000 to 250 Hz and filtered using 40 Hz low-pass and 0.01 Hz high-pass filters. For each participant, channels with excessive noise were identified by visual inspection and replaced via interpolation, using a weighted average of the surrounding electrodes. Data were then rereferenced by subtracting the mean activation across all electrodes from each individual electrode at each time point. Data were divided into epochs from −1 to +5 s from the onset of the reward cues. Epochs containing artifacts (e.g., muscle activity) were rejected based on visual inspection. In the final stage of preprocessing, data were subjected to an independent component analysis. Structured noise components, such as eye blinks, were removed, resulting in the dataset used for subsequent analyses. Before each analysis, data were *z* scored over the trial dimension and baseline corrected using a time window of 200–50 ms before the trial event of interest (e.g., cue or target presentation).

##### EEG analyses.

We used RSA (Kriegeskorte et al., 2008) to investigate how reward prospect influenced neural coding for different kinds of task-relevant information. There were two main advantages of using RSA to address this question. First, multivariate approaches leverage pattern information that would normally be averaged out in univariate analyses. This makes multivariate methods more sensitive to effects based on distributed patterns of neural activity (Kriegeskorte et al., 2006). Second, RSA allowed us to examine multiple, overlapping neural codes. In particular, the combination of RSA with models for different task variables provided a powerful method for separating neural coding of task rules, relevant and irrelevant target features, as well as motor responses. This ability to separate overlapping neural activity would be difficult to achieve with more traditional EEG methods, such as the analysis of event-related potentials.

In addition, we selected EEG for its high temporal resolution. Constraints on the temporal resolution of fMRI can make it challenging to isolate task rule coding from subsequent perceptual processing because the slow hemodynamic response can make it difficult to pinpoint effects in time and distinguish sustained anticipatory activity from transient stimulus-evoked responses. In contrast, high temporal resolution methods such as electroencephalography are needed because their ability to distinguish rapid stimulus-evoked dynamics makes them ideal for isolating the effects of reward on task rule coding from subsequent neural coding patterns.

The logic of our approach was to characterize neural coding patterns elicited by different trial conditions and test whether reward prospect led to more distinct task representations for the two tasks being performed (color vs shape judgements; Etzel et al., 2016; Westbrook and Braver, 2016). We were especially interested in the effect of reward on proactive control mechanisms, wherein goal-relevant information is encoded in preparation for upcoming task demands. This stands in contrast to reactive control processes, which serve to resolve task demands after their detection (Braver, 2012). Evidence of reward-modulated proactive control in our design would be seen as a difference in rule coding between high and low reward conditions, before target onset. Moreover, the difference in rule coding during this period should also be associated with improvements in behavioral performance (Etzel et al., 2016). Building on the results of Etzel et al. (2016), a central goal in the present work was to examine the effect reward prospect on rule coding during switch trials, when rules required flexible updating. Here we reasoned that switch trials involve the greatest interference between rule representations and thus reward prospect could improve flexible cognitive control (Shen and Chun, 2011; Kleinsorge and Rinkenauer, 2012) by increasing neural dissimilarity between rule codes. As a secondary aim, we performed theory-driven analyses that tested whether high reward leads to stronger neural coding for task-relevant compared with task-irrelevant target features, and whether relevant feature prioritization was associated with improved performance (Pessoa, 2017). Finally, we performed exploratory analyses that tested whether high reward modulated motor response coding and whether reward-induced changes in task coding were associated with downstream changes in sensorimotor processing. All analyses used data from all 61 EEG channels. While this limits inference about the regional sources of neural activity, it has the advantage of including all available data without any assumptions about source localization. Incorrect and omission trials were excluded from EEG analyses.

##### Neural coding across the trial.

**Figure 2.**
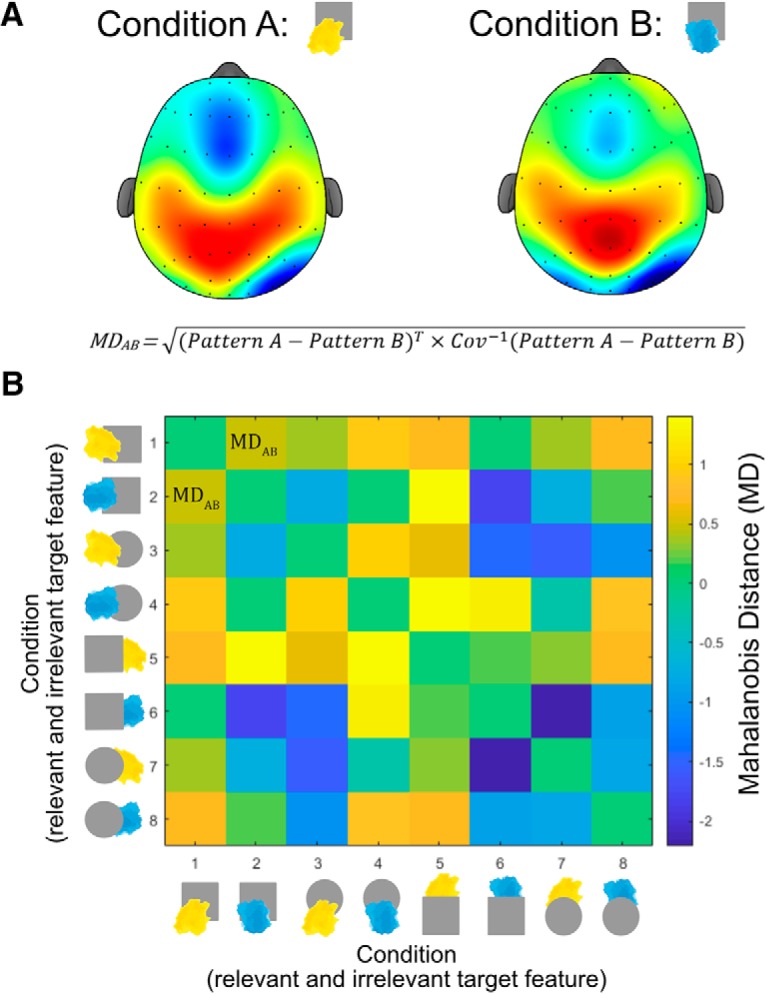
Logic of representational similarity analysis (RSA). ***A***, Trials were divided into conditions based on the relevant and irrelevant features of target stimuli. The dissimilarity between activity patterns was computed as the Mahalanobis distance (MD), which captures the multivariate distance between topographies. ***B***, MDs between conditions are entered into the relevant cells of a representational dissimilarity matrix (RDM). The process outlined in ***A*** is repeated until the MDs between each pair of conditions have been computed. The process is then repeated at the next time point and for subsequent time points of interest. The data RDM produced in ***B*** is then regressed against model RDMs that reflect predicted differences in dissimilarity structure for different task variables. For examples of model RDMs see Figure 4.

For each participant, trials were divided into conditions based on reward condition (low, high), as well as task-relevant and task-irrelevant target features. Dividing the trials this way also implicitly divided trials by task. If the task-relevant target feature was yellow, for example, then the task must have been to judge the target color. We then averaged trials in each condition to get an array with channels × time points × conditions. To measure neural dissimilarity between conditions, we used Mahalanobis distances (MDs). This distance metric shows similar reliability to correlation distance measures (Walther et al., 2016) but explicitly takes covariance into account, making it well suited to EEG data where channel values tend to be highly correlated. For EEG data, the MD between two conditions is computed at each time point, using two key pieces of information. The first is the difference in topographies between two conditions. A topography is a vector of 61 channel values that has been averaged across all trials in a condition, for a specific time point (Fig. 2*A*). The second key piece of information is the channel covariance matrix. This is computed using a matrix of trials × channels. For this study, we used within-condition error to compute the channel covariance matrix (Walther et al., 2016). This meant that before computing the covariance matrix, trials in the trial × channel matrix were mean centered by subtracting the mean topography for each condition from all trials within that condition. The covariance calculation also used a shrinkage estimator (Ledoit and Wolf, 2004), which has the effect of downweighting noisy covariance estimates. Using this information, the MD is formally computed as follows: MD_AB_ = $\sqrt{{\left(\text{Pattern}\;\text{A}-\text{Pattern}\;\text{B}\right)}^{T}\times {\text{Cov}}^{-1}\left(\text{Pattern}\;\text{A}-\text{Pattern}\;\text{B}\right)}$, where Pattern A − Pattern B is the difference between topographies, *T* is the transpose and Cov-1 is the inverse of the channel covariance matrix. This calculation is also presented in Figure 2. MDs were calculated between all topographies for the 16 conditions, separately at each time point. This procedure yielded a 16 × 16 representational dissimilarity matrix (RDM) of multivariate condition distances for each time point and participant. We then constructed a set of five 16 × 16 model RDMs to capture neural coding patterns related to different task variables. These variables included reward coding, task coding, task-relevant feature coding, task-irrelevant feature coding, and motor coding. The logic of all models was to place zeroes in cells of a 16 × 16 matrix where conditions matched on the variable of interest and ones in all remaining cells. For example, the task coding model was a 16 × 16 matrix containing zeros in cells where two conditions involved the same task (e.g., both color judgements) and ones in cells where two conditions involved different tasks (e.g., shape vs color judgements). The task-relevant feature model was a 16 × 16 matrix containing zeros in cells where two conditions had the same task-relevant target feature (e.g., both yellow on color trials) and ones in cells where two conditions had different task-relevant features (e.g., yellow vs blue or yellow vs square). Data RDMs (not *z* scored and averaged over time) and model RDMs for all task variables are presented for illustration (see Figure 4). For analysis, data and model RDMs were *z* scored. As RDMs are symmetric, the upper triangular portion of each matrix was transformed into a vector. The resulting data and model distance vectors were then entered into a multiple regression analysis that was conducted at each time point (4 ms apart after downsampling). The data-derived distance vector was the dependent variable and the model-derived distance vectors were independent variables. The regression also included a constant to model the intercept of the linear regression equation. This led to the following general linear model (where DV stands for distance vector):

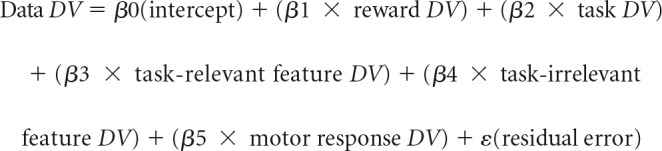
 The regression procedure was performed three times, once with a baseline window before reward cue onset, once before task cue onset and once before the target onset. Running the regression procedure at each time point produced a time course of regression coefficient estimates, one for each coding model. We refer to coefficient estimates as “model fit to neural dissimilarity matrix” in subsequent figures. We interpret the magnitude of these coefficient estimates to reflect the magnitude of neural dissimilarity/neural coding for each of the task variables across time.

##### Neural coding as a function of reward prospect.

We repeated the analyses above separately for high-reward and low-reward trials. For each participant, trials were divided based on the task-relevant and task-irrelevant features of the target. Conditions were then averaged over the trial dimension. Mahalanobis distances were calculated between all scalp topographies for the eight conditions, separately at each time point, generating a set of 8 × 8 RDMs. For these analyses, model RDMs were generated to reflect condition differences based on task coding, the task-relevant feature of the target, the task-irrelevant feature of the target, and the motor response. These models followed the same logic as those described in the previous section: zeroes were placed in cells of an 8 × 8 matrix where two conditions did not differ in the variable of interest (e.g., the task-relevant target feature), and ones were placed in cells where conditions differed in the variable of interest. Model and data RDMs were z scored. Like the previous section, RDMs were transformed into vectors and entered into a multiple regression (including a constant), that was conducted at each time point. The regression procedure was performed twice, once using only low-reward trials and once using only high-reward trials. For this set of analyses, it was particularly important to match the number of high-reward and low-reward trials, so that regression coefficients were not higher for high-reward conditions because more data were being included in the regression. To address this, these analyses subsampled trials to match the number of high-reward and low-reward trials. One hundred iterations were performed per participant, and final regression coefficients resulted from averaging the estimated regression coefficients over iterations. For task coding, we performed a theory-driven one-tailed *t* test to assess whether tasking coding was significantly greater under high-reward conditions during the pretarget period (averaged from 1400 to 1800 ms). This test was based on the results of Etzel et al. (2016) and remained significant when nondirectional. For reward-modulated coding of the relevant target feature, we tested whether the observed results could be driven by correlations with other coding models. To do so, we ran a control analysis in which we first regressed task coding, task-irrelevant feature coding, and motor coding against participants' neural dissimilarity matrices. We then regressed the task-relevant feature coding model against the residual variance, which had not been accounted for by the other models.

##### Neural coding as a function of rule updating.

We examined reward-modulated neural coding separately on switch and repeat trials. This was motivated by previous work showing that reward prospect benefits switching performance (Shen and Chun, 2011) and can exert a greater performance benefit on switch trials (Kleinsorge and Rinkenauer, 2012), where there is the highest interference between task sets and thus where neural separation between task rules could be particularly important in determining behavioral performance. These analyses followed the procedure outlined in the previous section but only used switch or repeat trials, instead of all trials.

##### Neural coding and cognitive performance.

To test relationships between neural coding and cognitive performance, we averaged regression coefficients over time windows of interest and correlated them with behavioral scores using nonparametric Spearman correlations. For task coding, we correlated the difference between regression coefficients averaged over the pretarget period (1400–1800) and the difference in switch costs between reward conditions. We undertook the same procedure for reward coding averaged from 0 to 5000 ms from the reward cue. To control for the influence of reward coding when correlating reward-modulated task coding with the changes in performance, we regressed out variance that could be explained by reward coding (averaged from 0 to 5000 ms) from the reward-task coding effect (high–low reward) and the reward-behavioral effect (e.g., RT low reward–RT high reward). We then correlated the residual variance from the reward-task coding effect (averaged over the pretarget period: 1400–1800 ms or the post-target period: 1800–2000 ms), with the residual variance in the reward-behavioral effect. We also tested whether the effect of reward on task coding as function of rule updating was correlated with the difference in switch cost between reward conditions. For this analysis, the reward-task sequence interaction term (see Fig. 6*C*) was averaged over the pretarget period (1400–1800 ms).

In addition to time-averaged correlations, we performed time-resolved brain–behavior correlations. To do this, we took participants' regression coefficients for different task variables at each time point along the trial and correlated this value with their difference in RT (low–high reward) and accuracy (high–low reward). Specifically, these analyses correlated a vector of 30 regression coefficient scores at a single time point with a vector of 30 behavioral scores. The same correlation approach was then performed at the next time point until a correlation had been performed at each time point along the window of interest. The false-positive rate for this time-resolved correlation approach was controlled using a cluster-based permutation procedure (detailed in Statistical testing for neural analysis), in which subjects' behavioral scores were randomly shuffled over many permutations. With the exception of reward coding (see Fig. 4*C*), which used the regression coefficient estimate directly, the neural data for these correlations were differences in regression coefficients between reward conditions. The behavioral data, such as the RT difference between reward conditions, were computed based on all trials used for the behavioral results section. In other words, these behavioral scores did not necessarily use identical trials used in EEG analyses, but instead made use of all behavioral trials available. The correlation analyses performed included neural data reflecting the difference in task coding (see Fig. 5*B*), relevant feature coding (see Fig. 8*A*), the interaction between reward and relevant feature prioritization (see Fig. 8*C*), as well as motor coding locked to the response (see Fig. 9*B*). Time-resolved correlations involving reward differences in neural regression coefficients used the same test windows as their corresponding figure, from which differences were computed. All analyses applied nonparametric Spearman correlations to reduce the influence of outliers in behavioral difference measures, which were more than three scaled median absolute deviations away from the median (RT difference distribution: two outliers, lower threshold = −42 ms, upper threshold = 68 ms, median = 13 ms; accuracy difference distribution: no outliers, lower threshold = −0.06, upper threshold = 0.10, median = 0.02).

##### Relationships among neural coding of task, sensory, and motor information.

To test whether reward-induced sensorimotor modulations arose from upstream changes in task coding, we performed a series of correlations between average task and sensorimotor coding regression coefficients. To do so, we selected time windows of interest based on results from the previous analysis section. For task coding, we averaged regression coefficients for each participant from 1400 to 1800 ms. We did not include 1200–1400 ms in this average due to a stimulus-evoked signal artifact shown in (see Fig. 5*B*). For feature coding and motor coding, we averaged regression coefficients from 2000 to 2400 ms, where we observed the peak difference between reward conditions (see Figs. 8*A*, 9*A*). For motor coding locked to the response, we averaged regression coefficients −200 to 0 ms from the response, within which we found the peak difference in motor coding between reward conditions. As initial tests, we correlated average task coding with average feature coding and average motor coding, independent of reward. Significant correlations from this first step were followed up by correlating the mean difference in task coding (high–low reward) with the mean difference in the relevant sensorimotor variable (high–low reward). All correlations were nonparametric Spearman correlations. The Bonferroni–Holm correction was used to correct for multiple comparisons (Holm, 1979). When using this procedure, corrected *p* values can become larger than 1. To avoid confusion, corrected *p* values larger than 1 were rounded to *p* = 1.

##### Statistical testing for neural analyses.

Data were smoothed with a 12 ms Gaussian kernel immediately before nonparametric cluster-based permutation testing, which was used to correct for multiple comparisons (Maris and Oostenveld, 2007; Spaak et al., 2017; Sassenhagen and Draschkow, 2019). This nonparametric approach is preferable to parametric statistical alternatives because it does not assume a particular distribution of the data. Cluster-based permutation testing computes a *t* statistic at each point in the observed data and many times in permuted data. Each permutation involves shuffling labels for the data, creating a permutated dataset that preserves temporal correlations within the EEG signal. Resulting *t* statistics are thresholded at an α-level of 0.05. Considering only the *t* values that remain, the cluster of time points with the largest absolute sum of *t* statistics (the largest cluster mass) from each permutation is placed into the null distribution. This null distribution shows the likelihood of obtaining a particular cluster mass due to chance. As a final step, candidate clusters from the observed data can then be compared against the null distribution. If the candidate cluster is larger than the 95th percentile of the null distribution, then the effect is considered significant at an α level of 0.05. Ten thousand permutations were performed to generate null distributions for each analysis in the present study.

## Results

### High reward decreased reaction time and improved accuracy

**Figure 3.**
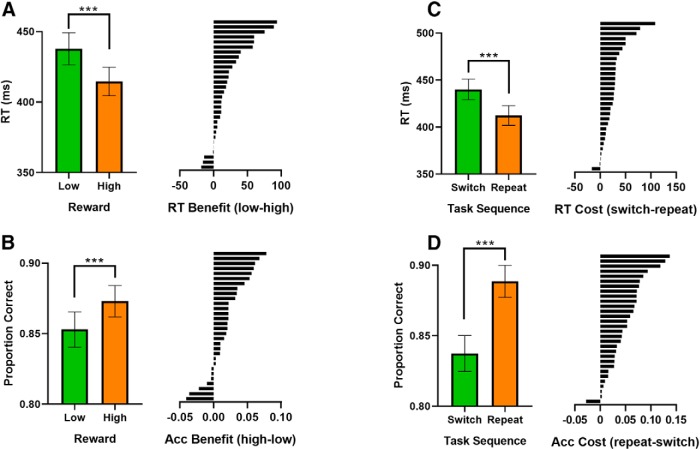
Behavioral performance as a function of reward and task sequence. ***A***, Reaction time for high-reward and low-reward trials (left) and the difference between reward conditions for each participant (right). ***B***, Proportion correct (accuracy) for high-reward and low-reward trials (left) and the difference between reward conditions per participant (right). ***C***, Reaction time as a function of task sequence (left) and the difference between switch and repeat trials for each participant (right). ***D***, Proportion correct (accuracy) as a function of task sequence (left) and the difference between switch and repeat trials (right) per participant. Error bars show the SEM. ***p < 0.001.

To assess the impact of reward prospect on behavioral performance, we performed a 2 × 2 repeated-measures ANOVA on RT, with factors of reward (low × high) and task sequence (switch × repeat). This revealed a significant main effect of reward (Fig. 3; *F*_(1,29)_ = 18.676, *p* < 0.001, η2*p* = 0.392), which reflected lower RTs on high-reward trials (mean = 415 ms, SD = 55.45) compared with low-reward trials (mean = 438 ms, SD = 62.28). There was also a significant effect of task sequence (*F*_(1,29)_ = 35.546, *p* < 0.001, η2*p* = 0.551), driven by lower RTs on repeat trials (mean = 412 ms, SD = 57.64) compared with switch trials (mean = 440 ms, SD = 59.36). While switch costs were numerically reduced on high-reward trials compared with low-reward trials, the interaction between reward and task sequence did not reach significance (*F*_(1,29)_ = 2.662, *p* = 0.114, η2*p* = 0.084). A control analysis comparing switch trials with repeat trials involving different task cues showed significantly higher RTs on switch trials, confirming that the main effect of task sequence was driven by genuine changes in task set and not simple changes in visual cues (Shapiro–Wilk test for normality: *W* = 0.822, *p* = 0.003; Wilcoxon signed-rank test: *T* = 438.0, *p* < 0.001, matched rank biserial correlation = 0.884).

The equivalent 2 × 2 ANOVA for proportion correct showed the same pattern of results. In particular, the analysis showed a significant main effect of reward (*F*_(1,29)_ = 14.268, *p* < 0.001, η2*p* = 0.330), driven by greater accuracy on high-reward trials (mean = 0.87, SD = 0.06) compared with low-reward trials (mean = 0.85, SD = 0.07). It also showed a significant main effect of task sequence (*F*_(1,29)_ = 51.894, *p* < 0.001, η2*p* = 0.642), reflecting greater accuracy for repeat trials (mean = 0.89, SD = 0.06) compared with switch trials (mean = 0.84, SD = 0.07). We did not detect a significant interaction between reward and task sequence (*F*_(1,29)_ = 0.317, *p* = 0.578, η2*p* = 0.011). Like RT, a control analysis comparing switch trials with repeat trials involving different task cues showed significantly lower accuracy on switch trials, confirming that the effect of task sequence on accuracy was driven by genuine changes in task set (Shapiro–Wilk test for normality: *W* = 0.977, *p* = 0.744; *t*_(29)_ = −6.110, *p* < 0.001, *d* = −1.116). To summarize, our behavioral analyses show that high reward prospect improved both RT and accuracy performance.

### Neural coding across the trial

**Figure 4.**
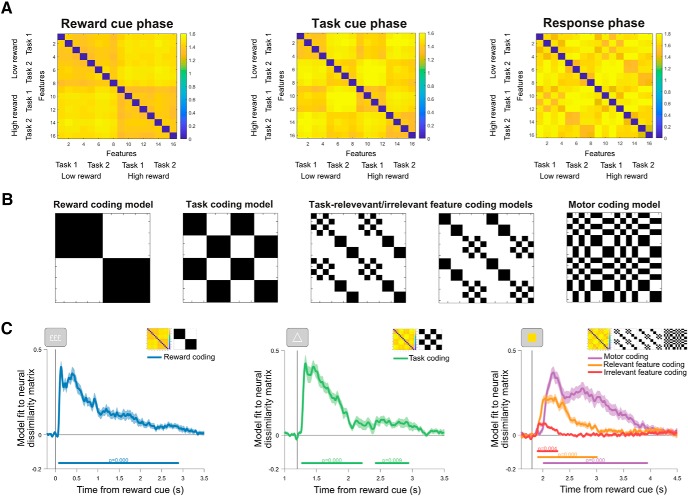
***A***, Representational dissimilarity matrices averaged across participants and trial periods following reward cue onset (0–800 ms), task cue onset (1200–1800 ms), and target onset (1800–2600 ms). ***B***, Model representational dissimilarity matrices for reward coding, task coding, coding of the task-relevant target feature, task-irrelevant target feature, and motor coding. ***C***, General linear model regression coefficients from regressing model and neural dissimilarity matrices across time. Shading around principle lines indicates SEM. Cluster-corrected *p* values are shown below each time course.

Having established the beneficial impact of reward prospect on cognitive performance, we tested the neural coding of task variables across the trial. Reward coding emerged shortly after reward cue onset and was sustained throughout the trial (Fig. 4; window tested = 0–3500 ms from reward cue onset, cluster window = 68–2940 ms, *p* = 0.0002). Task coding peaked shortly after the task rule cue was presented and continued into the response phase (window tested = 1200–3500 ms from reward cue onset; first cluster = 1260–2220 ms, first cluster *p* = 0.0002; second cluster = 2420–2948 ms, second cluster *p* = 0.0094). Finally, the coding of task-relevant and task-irrelevant target features, as well as motor response coding rose shortly after target presentation (relevant feature coding: window tested = 1800–4500 ms; cluster window = 1900–3016 ms; *p* = 0.0002; irrelevant feature coding: window tested = 1800–4500 ms from reward cue onset; cluster window = 1892–2284 ms; cluster *p* = 0.0036; motor coding: window tested = 1800–4500 ms; cluster window = 2004–3952 ms; cluster *p* = 0.0002). In summary, we verified that our multivariate analysis approach was sensitive to dynamic temporal changes in the neural coding of task variables at sensible stages within the trial.

### Task coding as a function of reward prospect

**Figure 5.**
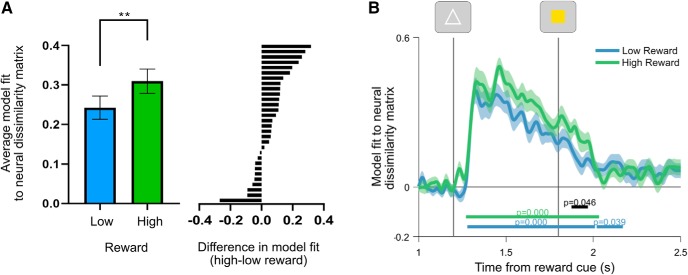
Generalized linear model regression coefficients for task coding as a function of reward. ***A***, coefficients averaged over time points in the pretarget interval (1400–1800 ms). The left subpanel shows group means for average regression betas as a function of reward. The right subpanel shows the difference between high-reward and low-reward regression coefficients for each participant. ***B***, Time-resolved regression coefficients for task coding as a function of reward. Vertical lines show onset of the task cue and target, respectively. Shading around principle lines indicates the SEM. Cluster-corrected *p* values are shown below time courses. **p < 0.01.

Reward prospect increased proactive task coding. Having verified that core task variables were encoded in the EEG signal at the plausible stages within the trial, we tested which variables were influenced by the reward manipulation. We found that average task coding was significantly greater on high-reward trials before target onset (Fig. 5*A*; window averaged = 1400–1800 ms; Shapiro–Wilk test for normality: *W* = 0.973, *p* = 0.633; theory-driven one-tailed test based on Etzel et al. (2016): *t*_(29)_ = 2.881, *p* = 0.004, *d* = 0.526). Time-resolved permutation analyses confirmed robust encoding of task rules before the target under both reward conditions (Fig. 5*B*; window tested = 1200–2500 ms; first low-reward cluster = 1280–2008 ms; low reward *p* = 0.0002; second low-reward cluster = 2020–2168 ms; high-reward cluster = 1272–2032 ms, high reward *p* = 0.0002; difference cluster = 1876–1968 ms; *p* = 0.0460).

### Task coding as a function of rule updating

**Figure 6.**
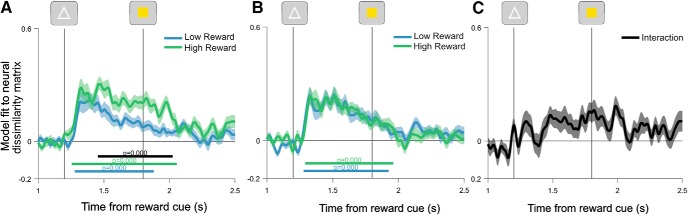
***A***, ***B***, Time-resolved regression coefficients for task coding as a function of reward on switch trials (***A***) and repeat trials (***B***). ***C***, Interaction between reward and task sequence regression coefficients. This time course is computed by taking the difference between task-coding regression coefficients on switch and repeat trials, within each reward condition. Resulting time courses are subtracted to show the extent to which high reward modulates the difference in task coding between switch and repeat trials. Vertical lines show the onset of the task cue and target, respectively. Shading around principle lines indicates the SEM. Cluster-corrected *p* values are shown below time courses.

Reward-induced increases in task coding were higher on switch trials. Having shown reward prospect-modulated task coding overall, we tested whether the effect of reward was amplified during rule updating, where interference between competing rules is highest and thus increased the separation between rules that could benefit flexible behavior. This analysis revealed a significant increase in task coding on high-reward switch trials compared with low-reward switch trials (Fig. 6*A*; window tested = 1200–2500; difference cluster = 1456–2024 ms; *p* = 0.0002). By contrast, we did not detect a difference in task coding as a function of reward on repeat trials (Fig. 6*B*; window tested = 1200–2500 ms; longest candidate cluster = 1224–1244 ms; *p* = 0.6825). The interaction between reward and task coding, as a function of rule updating, was significant when averaged over the pretarget interval (1400–1800 ms; *t*_(29)_ = 2.9247, *p* = 0.0066) and showed a trend toward significance when fully time resolved (Fig. 6*C*, window tested = 1200–2500 ms; longest candidate cluster = 1464–1544 ms; *p* = 0.0824).

### Task coding and cognitive performance

**Figure 7.**
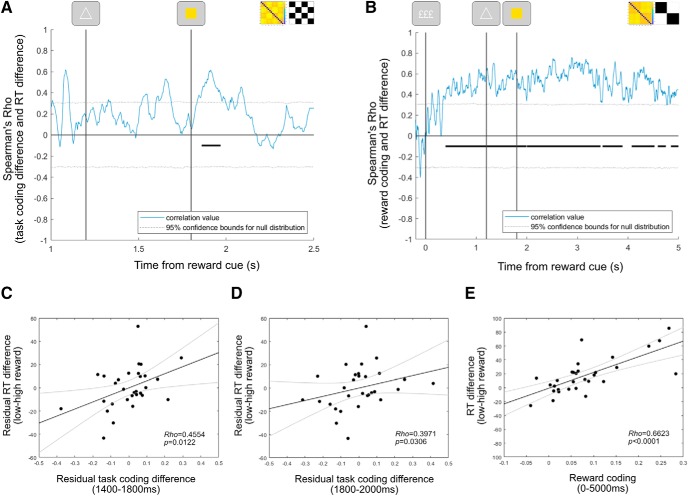
Relationships between reward-modulated task coding and performance. ***A***, Spearman's rho values for correlation between the difference in task-coding regression coefficients (high–low reward) and their difference in reaction time between reward conditions. ***B***, Spearman rho values for correlation between participant reward-coding regression coefficients and their difference in reaction time between reward conditions. In both ***A*** and ***B***, black lines indicate significant correlation clusters, corrected for multiple comparisons using cluster-based permutation testing. Gray dotted lines indicate 95% confidence intervals for the null distribution. ***C***, Scatterplot showing the relationship between the RT difference (low–high reward) and pretarget task coding (high–low reward, 1400–1800 ms), after removing variance associated with reward coding. ***D***, Scatterplot showing the relationship between the RT difference (low–high reward) and post-target task coding (high–low reward, 1800–2000 ms), after removing the variance associated with reward coding. ***E***, Scatterplot showing the relationship between the RT difference (low-high reward) and reward coding (0–5000 ms). In ***C–E***, black lines indicate linear fits to the data, and gray lines indicate 95% confidence intervals of the fits.

Neural encoding of task rules was associated with performance improvements. To understand the strong performance benefits observed in behavior, we tested whether task coding was associated with performance improvements (Etzel et al., 2016). The difference in task coding between reward conditions showed a significant correlation with reward-induced changes in RT (Fig. 7*A*; window tested = 1200–2500 ms; RT difference: cluster window = 1860–1968 ms; mean rho = 0.4927; *p* = 0.0225), but not with accuracy (longest candidate cluster = 2280–2320 ms; mean rho = −0.4083; *p* = 0.5382). Control analyses confirmed the relationship between reward-induced changes in task coding, and RT reflected the difference in RT between reward conditions, rather than either reward condition individually (window tested = 1200–1800 ms; high-reward RT only: longest candidate cluster = 1928–1948 ms; mean rho = −0.4004; *p* = 0.3030; low-reward RT only: longest candidate cluster = 1636–1664 ms; mean rho = 0.4602; *p* = 0.3502). We did not detect significant associations between the reward–rule updating interaction (Fig. 6*C*) and the change in switch costs between reward conditions for RT or accuracy (window averaged = 1400–1800; RT switch cost difference: Spearman's rho = −0.2725; *p* = 0.1449; accuracy switch cost difference: Spearman's rho = −0.3351; *p* = 0.0703).

Reward coding itself also showed a strong relationship with changes in performance. The magnitude of reward coding was significantly correlated with participants' difference in RT on high-reward compared with low-reward trials (Fig. 7*B*: window tested = 0–5000 ms; first cluster window = 392–1992 ms, mean rho = 0.5049, *p* = 0.0008; second cluster window = 2000–3456 ms, mean rho = 0.6001, *p* = 0.0004; third cluster window = 3496–3892 ms, mean rho = 0.5278, *p* = 0.0078; fourth cluster window = 4072–4524 ms, mean rho = 0.5483, *p* = 0.0100; fifth cluster = 4588–4748 ms, mean rho = 0.4487, *p* = 0.0415; sixth cluster = 4848–4996 ms, mean rho = 0.4112, *p* = 0.0459). Control analyses correlating reward coding with RT separately on high-reward or low-reward trials confirmed that this reward–coding association was specific to the difference in performance (window tested = 0–5000 ms; high-reward RT only: longest cluster = 1188–1248, mean rho = −0.4113, *p* = 0.3258; low-reward RT only: longest cluster = 4484–4512 ms, mean rho = 0.3939, *p* = 0.3656). We did not detect a significant correlation between reward coding and the difference in accuracy between reward conditions (window tested = 0–5000 ms; longest candidate cluster = 4–8 ms; mean rho = −0.3822; *p* = 0.7373). Similarly, we did not detect significant relationships between reward coding and the difference in switch cost between reward conditions for RT (window averaged = 0–5000 ms; Spearman's rho = 0.2912; *p* = 0.1185) or accuracy (window averaged = 0–5000 ms; Spearman's rho = 0.0472; *p* = 0.8045).

To test whether the relationship between reward-driven changes in task coding and improved RT performance was driven by reward coding itself, we ran a control analysis that regressed out variance associated with reward coding (averaged from 0 to 5000 ms) from the reward-task coding effect (high–low) and the reward-RT effect (low–high). We then tested the association between the residual variance in the reward-task coding effect and the residual variance in the reward-RT effect, using nonparametric Spearman correlations. This control indicated that reward-modulated task coding during the pretarget period (averaged from 1400 to 1800 ms) was associated with the change in RT performance, even after removing variance that could be accounted for by reward coding (Fig. 7*C*; Spearman's rho = 0.4554, *p* = 0.0122). This was also the case for reward-modulated task coding during the 1800–2000 ms post-target period (Fig. 7*D*; Spearman's rho = 0.3971, *p* = 0.0306). As a final control, we applied this procedure to cluster windows taken from Figure 7, *A* and *B* (task difference cluster range = 1860–1968 ms; reward cluster range = 392–4996 ms). This confirmed that the significant time-resolved correlation cluster identified for reward-modulated task coding (Fig. 7*A*) was associated with reward-driven changes in RT, independent of reward coding itself (Spearman's rho = 0.5075, *p* = 0.00469).

### Feature coding as a function of reward prospect

**Figure 8.**
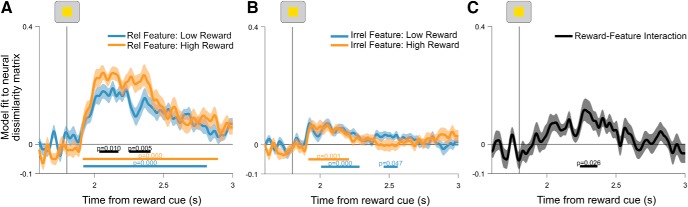
General linear model regression coefficients for coding of target features as a function of reward. ***A***, Task-relevant target feature regression coefficients. ***B***, Task-irrelevant feature regression coefficients. ***C***, Interaction between reward and feature regression coefficients. This time course is computed by taking the difference between task-relevant and task-irrelevant regression coefficients within each reward condition. Resulting time courses are subtracted to show the extent to which high reward increases the difference between task-relevant and task-irrelevant feature coding. Vertical lines show target onset. Shading around principle lines indicates SEM. Cluster-corrected *p* values are shown below time courses.

Reward prospect increased the coding of task-relevant target features. After evaluating the impact of reward prospect on task coding, we examined the effect of reward prospect on neural coding of task-relevant and task-irrelevant target features. Relevant feature coding was observed shortly after target presentation on both high-reward and low-reward trials (Fig. 8*A*; window tested = 1800–3000 ms; low-reward cluster = 1920–2812 ms, *p* = 0.0002; first high-reward cluster = 1916–2892 ms, *p* = 0.0002). In addition, neural coding for task-relevant features was significantly higher under high-reward conditions (window tested = 1800–3000 ms; first difference cluster = 2036–2172, *p* = 0.0100; second difference cluster = 2252–2404 ms, difference *p* = 0.0052). A control analysis that regressed the task-relevant feature model against the residual variance, which was not explained by any of the other coding models, confirmed that this result was not driven by correlated regressors (window tested = 1800–3000; low reward cluster = 1920–2812 ms, *p* = 0.0002; high-reward cluster = 1916–2892 ms, *p* = 0.0002; first difference cluster = 2036–2172 ms, *p* = 0.0120; second difference cluster = 2252–2404, *p* = 0.0058). Task-irrelevant information was also represented following target onset for both reward levels (Fig. 8*B*; window tested = 1800–3000 ms; low-reward cluster = 2008–2284 ms, *p* = 0.0002; second low-reward cluster = 2460–2560 ms, *p* = 0.0470; high-reward cluster = 1916–2208 ms, *p* = 0.0008). However, the strength of these coding patterns did not differ as a function of reward (window tested = 1800–3000 ms, no candidate clusters). As a consequence of these target-related effects, reward prospect showed a significant interaction with the difference in task-relevant and task-irrelevant feature coding. This reflected a greater difference between task-relevant and task-irrelevant coding under high-reward conditions (Fig. 8*C*; window tested = 1800–3000 ms; interaction cluster = 2240–2364 ms, *p* = 0.0258). In summary, we found evidence that high reward prospect increased the difference in neural coding for task-relevant and task-irrelevant target information. This difference was due to a selective increase in the neural coding of task-relevant feature information in high-reward contexts.

### Feature coding as a function of rule updating

Reward–feature coding modulations did not differ on switch trials. While high reward prospect increased the neural coding of task-relevant feature information overall (Fig. 8), the difference in relevant feature coding between reward conditions was not significant when switch and repeat trials were analyzed separately (window tested = 1800–3000 ms; switch trials: longest candidate cluster = 2144–2172 ms, *p* = 0.2274; repeat trials: longest candidate cluster = 2104–2148 ms, *p* = 0.1902).

### Feature coding and cognitive performance

Reward–feature coding modulations were not linked to performance improvements. In contrast to the relationships between task coding and performance improvements, we did not detect significant associations between reward-induced changes in feature coding and reward-induced changes in cognitive performance. This was the case for relevant feature coding (window tested = 1800–3000 ms; correlation with RT: longest cluster = 1880–1884 ms, mean rho = −0.3809, *p* = 0.8172; correlation with accuracy: longest cluster = 2660–2676 ms, mean rho = −0.3842, *p* = 0.6628), as well as the interaction between reward and relevant feature prioritization (Fig. 8*C*; window tested = 1800–3000 ms; correlation with RT: longest cluster = 1868–1880 ms, mean rho = −0.4230, *p* = 0.6568; correlation with accuracy: longest cluster = 2752–2764 ms, mean rho = 0.3789, *p* = 0.7073).

### Motor coding as a function of reward prospect

**Figure 9.**
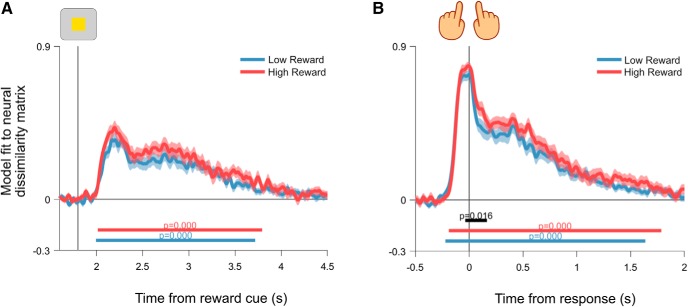
General linear model regression coefficients for coding of the upcoming motor response as a function of reward. ***A***, Motor model regression coefficients for data locked to reward cue onset. The vertical line indicates target onset. ***B***, Motor model regression coefficients for data locked to the response. The vertical line indicates the point at which responses were made. Shading around principle lines indicates the SEM. Cluster-corrected *p* values are shown below time courses.

Reward prospect increased the neural encoding of task-relevant motor output. Having established that reward prospect modulated neural activity coding for task-relevant target features, we examined the effect of reward prospect on activity patterns related to the upcoming motor response. When the analysis was locked to the onset of the reward cue, motor coding appeared after target presentation during both high-reward and low-reward conditions (Fig. 9*A*; window tested = 1800–4500 ms; low reward cluster = 1996–3716 ms, low reward *p* = 0.0002; high-reward cluster = 2016–3792 ms, high reward *p* = 0.0002) and showed a trend toward higher motor coding under high-reward conditions (Fig. 9*A*; window tested = 1800–4500 ms, difference cluster = 2040–2152 ms, *p* = 0.0900). When the analysis was locked to the onset of the motor response itself, we observed significantly higher motor coding on high-reward trials (Fig. 9*B*; window tested = −500 to 2000 ms from response; low reward cluster = −220–1636 ms, low reward *p* = 0.0002; high-reward cluster = −180–1784 ms, high reward *p* = 0.0002; difference cluster = −36–164 ms, *p* = 0.0158).

### Motor coding as a function of rule updating

Reward prospect modulated motor coding on switch and repeat trials. Based on the reward–motor coding effect observed in our response-locked analysis (Fig. 9*B*), we tested whether this effect differed on switch and repeat trials. Motor coding was significantly greater on high-reward compared with low-reward switch trials (window tested = −500 to 2000 ms from response, longest difference cluster = −72 to 180 ms, *p* = 0.0040), as well as high-reward compared with low-reward repeat trials (window tested = −500 to 2000 ms from response; first difference cluster = −84 to 120 ms, *p* = 0.0200; second difference cluster = 1512–1748 ms, *p* = 0.0240).

### Motor coding and cognitive performance

Reward–motor coding modulations were not linked to performance improvements. When testing whether the reward boost in motor coding (Fig. 9*B*) was associated with changes in performance, we did not detect a significant association between the increase in motor coding (high reward–low reward) and the change in RT between reward conditions (window tested = −500 to 2000 ms; longest difference cluster = −192 to −152 ms; mean rho = −0.4415, *p* = 0.3505). Similarly, we did not detect a significant association between the reward boost in motor coding and the change in accuracy between reward conditions (window tested = −500 to 2000 ms; longest difference cluster = 468–524 ms; mean rho = −0.4618, *p* = 0.1985).

### Relationships among neural coding of task rule, feature, and motor information

Reward-induced changes in task coding were not associated with sensorimotor effects. Having established that reward prospect modulated proactive coding of task information, we investigated whether such proactive changes could account for modulations in post-target processing. To do so, we first tested whether average task coding during the pretarget interval (1400–1800 ms) was associated with average feature coding (2000–2400 ms), as well as average motor coding locked to the reward cue (2000–2400 ms) and the response (−200 to 0 ms). Task coding was significantly correlated with coding of the relevant target feature (Spearman's rho = 0.5355, Bonferroni–Holm corrected *p* = 0.0156) and the difference between task-relevant and task-irrelevant features (Spearman's rho = 0.4719, Bonferroni–Holm corrected *p* = 0.0455), indicating that participants with greater task coding also tended to exhibit greater prioritization of task-relevant target features. We did not detect significant correlations between task coding and motor coding locked to the reward cue (Spearman's rho = −0.1511, Bonferroni–Holm corrected *p* = 1) or the response (Spearman's rho = −0.1448, Bonferroni–Holm corrected *p* = 1). Having established these relationships, we tested whether reward modulations in task coding during the pretarget period (high reward–low reward, 1400–1800 ms) were correlated with reward modulations in relevant feature coding following target onset (high reward–low reward, 2000–2400 ms). This analysis did not detect a significant correlation between reward-modulated task and reward-modulated feature coding (Spearman's rho = 0.1097, Bonferroni–Holm corrected *p* = 0.8870). The same pattern of results was found when testing the association between reward-modulated task coding (high reward–low reward, 1400–1800 ms) and the extent to which high reward increased the representation of the relevant target feature over the irrelevant feature (2000–2400 ms; Spearman's rho = 0.2957; Bonferroni-Holm corrected *p* = 0.4512).

## Discussion

The present study aimed to investigate how rewards modulate the neural coding of task rule information to support flexible cognitive control. We used RSA to examine changes in the neural coding of tasks as the prospect of reward changed dynamically from trial to trial. Using this method, we were able to track neural representations for reward prospect, task rules, task-relevant and task-irrelevant perceptual features of target stimuli, and neural representations related to accurate motor output. We found that high reward prospect boosted the encoding of multiple task variables. Critically, reward increased encoding of the active task rule in preparation for the target, and the extent of this increase was associated with reward-based reductions in RT. In addition, the modulatory effect of reward on rule coding was amplified on switch trials, where more flexible processing of rules was needed. Following the target, we observed increased encoding of task-relevant perceptual and motor information under high-reward conditions.

Consistent with the results of previous fMRI decoding studies (Woolgar et al., 2011; Waskom et al., 2014; Wisniewski et al., 2015; Etzel et al., 2016; Qiao et al., 2017), RSA successfully tracked neural representations for task rule information. In using this approach, our results replicate the fMRI findings of Etzel et al. (2016) in human electroencephalographic data, showing that high reward prospect increased average task coding before target onset, and that the difference in task coding between reward conditions was associated with improvements in cognitive performance. The effect of reward on task coding was most pronounced on switch trials, where task rules needed to be updated relative to the previous trial. This could suggest that dynamic increases in reward prospect primarily promote flexible rule updating, as opposed to maintenance of existing task rule representations in prefrontal regions. Such updating might be mediated by phasic dopamine release in the striatum, which is thought to be important for driving flexible and targeted updating of contextual information in PFC (Westbrook and Braver, 2016; Yee and Braver, 2018). In light of our neural results, showing a larger reward effect on switch trials, it is surprising that there was not a significant corresponding effect in behavior. We believe that this behavioral result should be interpreted with caution, as there was a clear trend in the expected direction, and previous studies have observed robust reward effects on rule switching (Shen and Chun, 2011; Kleinsorge and Rinkenauer, 2012). Overall, the present results provide evidence that high reward prospect can improve cognitive performance by increasing proactive neural representations for task rule information. Moreover, these results seem to be consistent with the theoretical view that reward prospect could help separate the representations of competing task rules, which is especially critical during transitions between different behavioral contexts (Waskom et al., 2014). Indeed, such a mechanism could account for the selective effects of reward on switching that has been observed in previous studies (Shen and Chun, 2011; Kleinsorge and Rinkenauer, 2012).

One interesting aspect of these results was that reward-driven changes in task coding were primarily associated with improvements in RT but not in accuracy. This result is distinct from the findings of Etzel et al. (2016), who found that reward-driven increases in task coding mediated improvements in accuracy but not in RT. A simple explanation for this could be our RT incentive manipulation, which made RT a more informative behavioral measure in the current study. On top of this, RT values have a wider dynamic range than accuracy, which could have made it easier to detect associations between neural measures and RT performance. From a theoretical perspective, the asymmetry between our RT and accuracy correlation results might be viewed as an optimal control problem, in which subjects maximize expected value over time through balancing speed and accuracy (Bogacz, 2007; Manohar et al., 2015). In our task, accuracy rates were relatively high, and, while reward significantly increased accuracy, the effect was much smaller than for RT. One possible explanation for this comes from Manohar et al. (2015), who propose an optimal control model to account for the effects of reward on neural noise and behavioral performance. One important prediction of this model is that when the signal-to-noise ratio in a neural system is high, rewards will have a disproportionate effect on response vigor and the reduction of RT. These signal-to-noise conditions might well have reflected our task conditions, in which accuracy rates were high and stimuli were unambiguous.

Consistent with the results from previous studies focusing on neural responses to perceptual targets (Serences, 2008; Serences and Saproo, 2010; Padmala and Pessoa, 2011; Hickey and Peelen, 2015), we found that reward enhanced the representation of task-relevant perceptual features. The present results complement this literature in two ways. First, many previous studies have focused on perceptual stimuli associated with reward over many trials (Serences, 2008; Serences and Saproo, 2010; Hickey and Peelen, 2015). Here we show that transient changes in prospective reward can also modulate task-relevant perceptual feature representations. Second, research using prospective reward cues has led to the proposal that reward motivation might benefit attentional filtering, either by enhancing task-relevant perceptual representations or suppressing task-irrelevant representations (Pessoa, 2017). Hickey and Peelen (2015) found evidence for both of these mechanisms, showing that reward could either enhance or suppress perceptual representations as a function of task relevance. In the present study, we report a more selective effect on perceptual representations, wherein high reward prospect increased the neural representation of task-relevant information without impacting the representation of irrelevant information. This might suggest that transient reward coupling with sensory target features has a different impact on perceptual encoding than reward associations established over many trials.

While task coding and the prioritization of task-relevant target features were strongly correlated, we did not find evidence that reward-driven modulations in these variables were associated. We are cautious not to overinterpret these null effects. These results do not rule out the possibility that reward-modulated task coding affects downstream perceptual representations. However, they do raise the possibility of an alternative mechanism, wherein reward prospect acts independently on multiple neural variables. Among these variables, our results suggest that neural encoding of reward prospect and task rule information are important factors associated with dynamic shifts in improving performance. The present results do not permit conclusive interpretations about the functional role of reward-driven perceptual and motor changes; although we did not detect significant associations between perceptual representations and behavioral measures, this does not imply that these variables were functionally irrelevant to task performance.

How might reward motivation translate into performance improvements more broadly? Previous studies have pointed to the idea that reward motivation might upregulate attention (Pessoa and Engelmann, 2010; Padmala and Pessoa, 2011; Etzel et al., 2016). For instance, reward and attention have been shown to recruit overlapping frontoparietal control regions (Pessoa and Engelmann, 2010) and have analogous effects on electrophysiological signatures of task preparation (van den Berg et al., 2014). One possibility in our study is that reward prospect had additional effects that were not captured by the conditions of our task. For instance, high reward prospect could have increased alertness and temporal attention to information proximal to reward cue presentation. This may explain why associations between behavioral measures and downstream reward effects, such as target feature and motor representations, could be noisier and less reliable than the strong correlation between behavior and reward itself.

To conclude, previous work has shown that reward motivation may improve cognitive performance by boosting the neural coding of task rules (Etzel et al., 2016). Here we demonstrate that high reward prospect can increase proactive coding of task rule information and that this proactive effect has a strong relationship with performance improvements. In addition, the effect of reward on rule coding was heightened during switch trials, where task rules were updated relative to the previous trial. This suggests that reward prospect might optimize flexible control processes by increasing the neural separation between task rules.
